# Prognostic Value of Serial Lactate Measurement in Pediatric Cardiac Surgery Patients with Congenital Heart Disease in Southeast Mexico

**DOI:** 10.3390/medsci14010035

**Published:** 2026-01-09

**Authors:** Ely Sanchez-Felix, Amonario Olivera-Mar, Miguel Santaularia-Tomas, Joan Johnson-Herrera, Laura Ortiz-Vera, Adrian Perez-Navarrete, Marcos Rivero-Peraza, Nina Mendez-Dominguez

**Affiliations:** 1 Hospital Regional de Alta Especialidad de la Peninsula de Yucatan, Servicios de Salud del Instituto Mexicano del Seguro Social para el Bienestar (IMSS-BIENESTAR), Merida 97300, Mexico; ely_felix15@hotmail.com (E.S.-F.); amar.hraepy@imssbienestar.gob.mx (A.O.-M.); msantaularia@imssbienestar.gob.mx (M.S.-T.); lauraortizv3@hotmail.com (L.O.-V.); prz.adrian@gmail.com (A.P.-N.); 00380342@anahuac.mx (M.R.-P.); 2 School of Medicine, Anahuac University, Km 15.5, Carr. Mérida-Progreso, Mérida 97302, Mexico

**Keywords:** prospective studies, congenital heart defects, cardiac surgical procedures, intensive care units

## Abstract

Background/Objectives: Lactate, traditionally considered a byproduct of anaerobic metabolism, is increasingly recognized as a biomarker of tissue perfusion and systemic stress. While hyperlactatemia is frequent after pediatric cardiac surgery, evidence regarding its prognostic role remains controversial. This study aimed to evaluate whether serial lactate measurements predict mortality in children undergoing surgery for congenital heart disease in Southeast Mexico. Methods: We conducted a retrospective cohort study including children aged 0–210 weeks with confirmed congenital heart disease who underwent first-time cardiac surgery between January 2022 and December 2024. Serum lactate was measured intraoperatively, at intensive care unit (ICU) admission, and at 12 and 24 h postoperatively using a Gem^®^ Premier™ 3500 analyzer. Sociodemographic, clinical, and surgical data were recorded. Associations between lactate levels and mortality were analyzed with Cox regression, adjusting for RACHS-2 category and intraoperative complications. Predictive performance was assessed with ROC curves and Harrell’s C-index. Results: 103 patients were included (median age 49.2 weeks; 60% female). Lactate levels overlapped intraoperatively but significantly discriminated against survivors from non-survivors thereafter. ICU admission lactate ≥ 4.2 mmol/L predicted mortality with 100% sensitivity and 60% specificity (AUC = 0.84). Hazard ratios confirmed that lactate at ICU admission (HR 2.17, 95% CI 1.16–4.06; *p* = 0.015), 12 h (HR 6.37, 95% CI 1.02–39.6; *p* = 0.047), and 24 h (HR 1.81, 95% CI 1.07–3.09; *p* = 0.028) were significant predictors of mortality. The model showed excellent discrimination (Harrell’s C = 0.986), though optimism due to the limited number of deaths should be considered. Conclusions: Serial lactate monitoring, particularly upon ICU admission, provides strong prognostic information for in-hospital mortality in pediatric cardiac surgery patients. Incorporating early postoperative lactate into routine monitoring may allow timely therapeutic adjustments. Preoperative lactate assessment warrants further evaluation as a potential risk stratification tool.

## 1. Introduction

The period surrounding pediatric cardiac surgery is critical for a child’s future well-being and quality of life, significantly impacting every family member. This crucial time is marked by clinical uncertainty and necessitates close monitoring [1]. However, even with diligent monitoring, establishing a patient’s prognosis is not always straightforward for the medical team. In this context, lactate research may offer insights and a measurable biomarker to guide treatment adjustments for patient stabilization.

L-lactate, the biochemical isomeric conjugated base of lactic acid, is clinically important due to its potential as a prognostic biomarker. Approximately 10–12% of patients develop hyperlactatemia after cardiac surgery [2,3]. Shock is characterized by elevated lactate levels, resulting from anaerobic metabolism when oxygen delivery is impaired. Large series of post-cardiac surgery patients consistently show that lactate predicts mortality within similar confidence ranges [4]. According to Minton & Sidebotham [5], early hyperlactatemia after adult cardiac surgery significantly predicts adverse outcomes, including hypoxic and non-hypoxic shock, while later-onset hyperlactatemia tends to resolve within hours. However, Zeng and colleagues found that lactate was not superior to lactate dehydrogenase when retrospectively compared in patients from the MIMIC III & IV database [6].

The pathophysiology of ischemia and hemodynamic homeostasis in neonates, infants, and toddlers with congenital heart conditions, with or without coexisting congenital conditions, should not be assumed to be congruent with that of adult patients. In 1997, Hatherill and colleagues dismissed the value of early lactate levels as mortality predictors due to significant overlapping values between survivors and non-survivors, though controversy persisted [6]. In 2023, Matsushita, Krebs & de Carvalho conducted a systematic review and meta-analysis to evaluate serum lactate as a predictor of morbidity and mortality in critically ill neonates from various causes. They concluded that, for cohort-based data, lactate testing is not recommended for predicting morbidity and mortality in neonates, but they also proposed developing serial measurements [7].

Besides its role as a marker indicating impaired tissue oxygenation, lactate has can act as a signaling molecule with a direct effect on immunometabolism. Fang et al. [8] have described how lactate modulates inflammatory mechanisms through histone lactylation and HIF-1α activation among other possible pathways, influencing both acute and chronic inflammatory responses. Inflammation itself can be strongly independently associated with adverse outcomes across critical care populations regardless of underlying conditions. Karagoz et al. [9] found that a higher Prognostic Nutritional Index, which is an integrative marker of nutritional and immunological status, was associated with lower mortality and had an inverse correlation with inflammation. Additionally, Yang et al. [10] reported that inflammatory biomarkers predicted both in-hospital and short-term mortality after acute myocardial infarction, and Liu in 2025 [11] demonstrated that systemic inflammation partly mediated the association between sarcopenia and mortality in older adults. These studies converge to support the hypothesis that inflammation represents a key pathway linking metabolic alterations, including hyperlactatemia, with poor outcomes, thereby providing additional rationale for evaluating lactate as a prognostic biomarker in pediatric cardiac surgery.

Given that neonates with diverse health conditions were included in such research, and the ongoing controversy, the objective of the present study is to analyze serial measurements of serum lactate after cardiac surgery in pediatric patients (birth to 210 weeks old) with congenital heart diseases as a predictor for survival or death. Existing literature assumes that the same lactate measurement timing periods apply for outcome prediction in both adult and pediatric patients, irrespective of ethnicity or sex. For our study, we included four timings from intraoperative to 24 h post-surgery.

## 2. Materials and Methods

### 2.1. Study Design and Participants

Through a retrospective cohort study in a tertiary care hospital in southeastern Mexico, analysis was conducted to determine the characteristics and prognostic factors after congenital heart disease repair. Children were included if their surgery was scheduled between January 2022 and December 2024. The inclusion criteria were confirmed congenital heart defects undergoing first-time cardiac surgery. Patients were followed in outpatient clinics up to July 2025. Patients who were programmed for reintervention in follow-up were excluded. Patients who did not have complete lactate monitoring or diagnosis at hospital discharge or were transferred to another health institution were eliminated from analysis. When sociodemographic information could not be obtained, datum was treated as missing.

### 2.2. Sample Size

For an estimated number of events of n = 103, a power of 0.94 was calculated with a significance level of α = 0.05 (two sided) for Cox regression.

### 2.3. Variables & Statistical Analysis

The baseline study variables were: (a) age in weeks as a continuous numerical variable, (b) male sex as a categorical dichotomous variable, (c) ethnicity as a categorical variable, patients’ parents were categorized as indigenous when surnames were in Mayan or from other native human group as specified, and (d) lactate values in Mmol as continuous numerical data at four different moments: 1. At the surgery while in the operating room on-pump, 2. at admission to the intensive care unit, (within the first 30 min after admission at ICU), 3. twelve hours after surgery, and 4. twenty-four hours after surgery. We also included the classification considering the main diagnosis using international Statistical Classification of Diseases (CDI) -10 codes (Q21.0, Q21.1, Q21.2, Q22.0, Q25), and risk using Risk Adjustment for Congenital Heart Surgery second edition (RACHS-2) scale, to stratify patients given their diagnosis; the scale categorizes surgical procedures into different risk categories, typically from 1 (lowest risk) to 6 (highest risk), based on their associated in-hospital mortality rates. After careful review, patients were classified considering if they had a Critical Congenital Heart Disease and if the condition was classified as cyanotic; also, we registered patients who received Extracorporeal membrane Oxygenation. Surgery-related variables also included intraoperative complication and hypothermia; all these as dichotomous variables.

Data on each surgical procedure to repair congenital heart diseases in pediatric patients were codified, anonymized, and entered into a spreadsheet. Categorical data are presented as total counts with frequencies and percentages. Numerical variables are presented as medians. Lactate was measured in mmol/L using a Gem^®^ Premier™ 3500. Values are presented grouped by sex, ethnicity, and age. For contrasting mean lactate values by ethnicity and sex, chi-square tests were performed, also for comparing between patients who were discharged due to improvement and those who died in hospitalization. For establishing correlations between age and lactate, and between the four timings of measurements, correlations were used. Comparative ROC analysis was performed, obtaining the area under the curve at different cutoff points for each timing of lactate measurement. Graphics were obtained along with specificity, sensitivity, diagnostic predictive value, false positive and false negative proportion at each cutoff point for the lactate timing exhibiting the most significant predictive value. For establishing associations between lactate and risk for mortality, a Cox regression model was performed expressing Hazard ratios, which were estimated to further identify the timing for the strongest prediction. For all analyses, Stata 19 software was employed, and in all tests, statistical significance was established at *p* < 0.05.

The study was approved on 9 December 2022 by the Institutional Review Board of Ethics and Research 2022-016. Patients’ legal guardians provided written informed consent for their children to participate in the study.

## 3. Results

From January 2022 to December 2024, 103 first-time cardiac surgeries were performed for repairing congenital heart diseases in pediatric patients. Age of patients ranged between birth and four years; forty were male. None of the patients or their families had affiliation with any medical insurance. Place of residence was the Yucatan peninsula located in southeast Mexico for all cases. Regarding ethnicity, 41 had fathers identified as Indigenous (Mayan), 38 were born to Indigenous mothers, and overall, 52 patients were Mayan and 51 were not, as presented in Table 1.

### 3.1. Descriptive Statistics

From January 2022 to December 2024, 103 first-time cardiac surgeries were performed for repairing congenital heart diseases. Age ranged between birth and four years; forty were male. None of the patients or their families had affiliation with any medical insurance. Place of residence was the Yucatan peninsula located in southeast Mexico for all cases, but 66% were residents of Yucatan, the state where the hospital is located, while 7% resided in Campeche and 17% in Quintana Roo.

### 3.2. Lactate Measurement

Values regarding lactate after surgery, at admittance at the Intensive Care Unit, 12 and 24 h after surgery are presented in Table 2, along with intraoperative characteristics.

Age did not correlate with lactate levels at any measurement, and between the timing of measurements, 12 and 24 h lactate correlated at R = 0.82 in the overall sample, and no other significant correlations were identified. Alpha Cronbach test reported a covariance of 1.62 with a scale reliability coefficient of 0.71 for survivors and 2.71, 0.90 for non-survivors, respectively.

**Figure 1:**
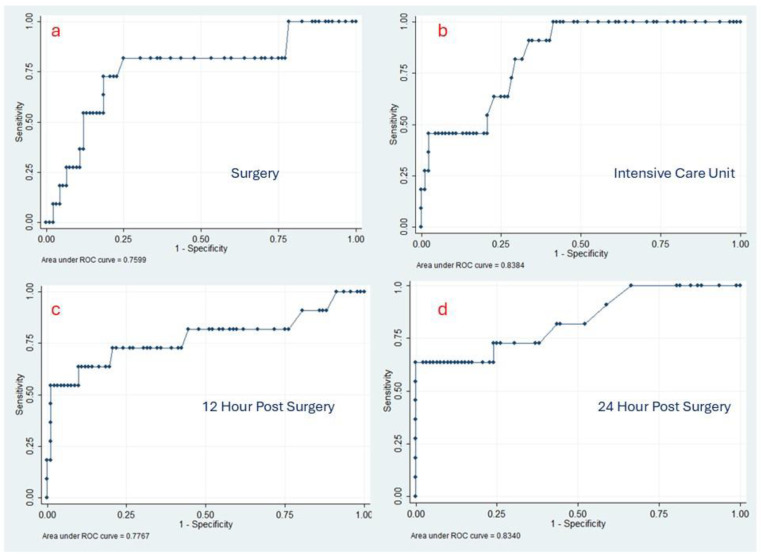
ROC curves indicating predictive value of mortality using lactate determination at four different moments in 103 pediatric patients with congenital heart disease. From left to right, (**a**) after the surgery while in the operating room on-pump, (**b**) within the first 30 min after admission at ICU, (**c**) twelve hours after surgery, (**d**) twenty-four hours after surgery.

Figure 1 presents the comparison of specificity, sensitivity, and area under the curve of lactate levels for predicting mortality at four different timings. Lactate values at admittance to the Intensive Care Unit exhibited a predictive value closer to 1, with an area under the curve of 0.84 in scale; at that timing of lactate determination, a value ≥4.2 would provide a 100% sensitivity and 60% specificity for mortality, allowing surgical team and intensive care staff to implement adjustments and prepare to assist patients with optimal care. 

Two group mean comparison tests showed that lactate levels at admittance to ICU was of 2.79 for patients who improved and 8.54 for patients who died (t = 5.75, *p* < 0.01). Lactate values at different timings showed a 0.83 Cronbach alpha correlation.

In Cox regression, hazard ratios adjusted by observed covariates indicated that lactate determination at admittance, 12 and 24 h post-surgery predicted mortality significantly, with the first mentioned determination being the most significant, but intraoperative was not statistically significant. The regression model demonstrated excellent discrimination, with a Harrell’s C-index of 0.986, indicating that in 98.6% of comparable patient pairs, the patient predicted to be at higher risk died. This extremely high value suggests strong predictive separation, though it should be interpreted with caution given frequency of fatalities (Table 3).

## 4. Discussion

In the present research, we addressed the controversy surrounding the use of serial lactate measurements versus a single determination for predicting adverse outcomes in pediatric patients following cardiac surgery for congenital heart disease repair; in retrospective, we found that even when intraoperatively measured, lactate did not significantly predict mortality. The serial measurements during monitorization are relevant to orient postsurgical therapeutic approaches.

In contrast to the findings reported by Hatherill and colleagues [7], the lactate determination after surgery only showed overlap in the initial measurement. Subsequently, the lactate ratio between patients who died and those who survived increased to over 4.28 in the latest measurement. Nevertheless, we concur with these authors that a single determination should not be considered sufficient for prognosis estimation. Furthermore, guided by the results of Matsushita and colleagues, we plan to implement pre-surgery determination of lactate, as it may serve as a useful biomarker of pre-surgical status [12].

Ranucci and colleagues implemented lactate determination every 10 min during surgery, finding that even after accounting for other intra-surgical biomarkers, peak lactate remained the only independent factor for mortality [13]. This is consistent with previous descriptions that impaired hepatic perfusion can lead to a decline in hepatic ability to utilize lactate, potentially causing the liver itself to produce lactate [14]. The severity of congenital heart disease may also influence lactate levels, even prior to surgery. Kapoor and colleagues conducted a prospective study of 150 patients, aged 6 months to 12 years old, with tetralogy of Fallot, measuring lactate before and after surgical repair; they observed significant differences in lactate levels prior to surgery, no difference at 20 min post-protamine measurement, and again significant values 24 h post-surgery. This suggests that patients may be at risk even before surgery.

In a series of 1355 patients aged 1–17, Nygaard and colleagues found no predictive value for adverse outcomes, including resuscitation, with lactate determination. They also reported an inverse correlation between age and lactate levels [15,16]. However, in our study, no significant correlation was found. This discrepancy could be explained by the wider age range in the Copenhagen University Hospital study compared to ours, and because their study included patients with various diagnoses.

Another relevant study by Mir et al. in 2006 [17] investigated the utility of N-terminal brain natriuretic peptide (N-BNP) plasma concentrations in comparison to lactate and troponin T in children following open-heart surgery for congenital heart disease. Their prospective study, involving 23 children, revealed that while N-BNP was correlated with postoperative vasodilator dosage and indicative of ventricular function, its value for guiding perioperative therapy in pediatric cardiac intensive care units was limited. Notably, their findings underscored the importance of lactate (aL) and troponin T (TnT), as these markers were significantly correlated with the duration and dosage of catecholamines and the duration of respiratory therapy. Kapoor and colleagues in 2016 predicted mortality in patients with congenital cardiopathy but restricted their observations to Tetralogy of Fallot carriers [16]. This contrasts with N-BNP, which did not show such correlations. Mir et al. (2006) suggest that a combination of a necrosis marker (troponin), an acidosis marker (lactate), and a volume/pressure marker (N-BNP) could be useful for a more comprehensive risk stratification of patients before and during the postoperative state [17].

Our findings emphasize the strong predictive value of serial lactate measurements, particularly at ICU admission, for in-hospital mortality. These findings are complemented by the work of Mir et al. While our study specifically focuses on the optimal timing and predictive power of lactate, their research reinforces lactate’s established utility as an acidosis marker that correlates with markers of clinical severity, such as catecholamine and respiratory support requirements. This combined perspective suggests that while lactate remains a crucial standalone indicator for immediate therapeutic adjustments, integrating it with other biomarkers like troponin and natriuretic peptides could offer a more holistic understanding of a patient’s physiological state and contribute to enhanced risk stratification and personalized critical care management.

We also found that sociodemographic constraints are also relevant in the context of the population we assist in southeast Mexico; more than a half of the children are parented by Mayan/mestizo, but we did not find differences in outcomes in relation to ethnicity. However, we found an increased percentage of patients from neighboring states of Campeche and Quintana Roo among those patients who did not survive, and far from thinking of undeclared propensities, we want to acknowledge how our study reinforces that geographic disparities in access to pediatric cardiac surgery are contextual factor to interpret our findings. In Latin America, specialized healthcare facilities are concentrated in a few large urban centers, creating structural barriers for children from rural or peripheral regions. Trujillo et al. (2025) identified geographic distance, financial hardship, and referral inefficiencies as major obstacles to timely pediatric care, disproportionately affecting families outside metropolitan areas [18]. Di Sessa et al. (2010) further estimated that approximately 24,000 children with congenital heart disease in South America remain untreated each year, largely due to unequal distribution of surgical centers and specialists [19]. Likewise, Vervoort et al. (2020) reported that in many Latin American countries, the density of pediatric cardiac surgeons is as low as 0.08 per million inhabitants, underscoring the scarcity of expertise beyond capital cities [20]. These inequities in access are likely to contribute to adversities and limit these children’s opportunities, and it is a matter of social justice, policies, and decision making in our changing health system.

The present study had several limitations that warrant acknowledgment. Firstly, a lack of preoperative risk stratification could have been performed with preoperative lactate. Preoperative physiological conditions and intraoperative parameters such as oxygen saturation, anemia, cardiopulmonary bypass and cross-clamp times were not recorded. These unmeasured confounders could partly explain the association between lactate levels and poor outcomes, and caution should be exercised in inferring causality. We focused exclusively on in-hospital mortality. Long-term morbidity, neurodevelopmental outcomes, and rehospitalization rates were not assessed but should be addressed in future prospective studies. Finally, given the underrepresentation of fatal cases for Cox regression we did not perform internal calibration analyses (e.g., bootstrap-corrected calibration slope), which should be addressed for larger cohorts.

## 5. Conclusions

From our retrospective cohort, we conclude that the most reliable timing for lactate determination in the post-surgical period in pediatric patients with congenital heart diseases is upon admission to the intensive care unit. This timing exhibited more significance, predictive value, and 100% sensitivity at ≥4.2 Mmol. Serial lactate determination should be employed, and pre-surgical lactate should be further explored for cardiac surgery in pediatric patients with congenital heart disease as an independent indicator for decision-making and help prepare the medical team for approaching a patient who could be at higher risk for adverse outcomes. Future studies should consider adding preoperative lactate determination to lactate monitorization.

## Figures and Tables

**Table 1:** Sociodemographic characteristics of 103 pediatric patients with confirmed congenital heart disease from a retrospective cohort.

Characteristics	General	Survivors	Non-Survivors
	**N = 103**	**N = 92**	**N = 11**
Mean age (weeks)	49.20	49.30	50.10
Percentage			
Maternal Mayan Surname	39.30	37.25	63.10
Paternal Mayan Surname	36.97	38.25	27.25
Mayan Ethnicity from either parent	50.57	47.89	72.76
Female	60.28	59.8	63.69
Male	39.89	40.20	36.40
Previous hospitalization	33.01	35.95 *	0
Reside in neighboring state	33.92	23.28 *	37.04

* Significant at *p* < 0.05.

**Table 2:** Clinical and surgical characteristics of 103 pediatric patients with confirmed congenital heart disease from a retrospective cohort.

Characteristics	General	Survivors	Non-Survivors
	N = 103	N = 92	N = 11
Days of hospital Stay	16.65	16.66	16.54
Minutes of Extracorporeal Membrane Oxygenation	104.70	76.26	156.12
Percentage			
Lactate At Surgery (mmol/L)	4.10	3.90	6.40
Lactate at ICU Admittance (mmol/L)	4.80	4.30	9.20 *
Lactate 12 Hours Post-Intervention (mmol/L)	3.30	2.80	8.50 *
Lactate 24 Hours Post-Intervention (mmol/L)	2.30	1.60	8.00 *
Q21.0: Ventricular Septal Defect	34.00	38.0	0
Q21.1: Atrial Septal Defect	47.6	46.7	54.50
Q21.2: Atrioventricular Septal Defect	8.7	7.60	18.20
Q22.0: Pulmonary Valve Atresia	8.7	7.60	18.20
Q25: Congenital Malformations of Great Arteries	1.00	0	9.10
Critical Congenital Heart Disease	16.5	13.00	45.50
Cyanotic Congenital Cardiopathy	17.5	14.10	45.50
Hypothermia	44.66	44.56	45.67
Intraoperative complications	27.18	26.08	36.36
Extracorporeal Membrane Oxygenation	23.22	49.14	36.54

* Significant at *p* < 0.05. Risk Ratio from lactate levels was between survivors and non-survivors, of 1.36 at admittance to ICU; 2.32 and 4.28 after 12 and 24 h, respectively.

**Table 3:** Regression analysis predicting mortality from serial lactate values in a sample of pediatric patients who underwent cardiac surgery.

Predictor	Hazard Ratio	Standard Error	z	*p*	95% Confidence Interval	
Lactate Value						
Intraoperative	0.78	0.20	−0.99	0.324	0.47	1.28
Intensive Care Unit Admittance	2.17	0.69	2.43	0.015 *	1.16	4.06
12 h	6.37	5.94	1.99	0.047 *	1.02	39.59
24 h	1.81	0.49	2.19	0.028 *	1.07	3.09
Observed Covariates						
RACHS II	0.27	0.21	−1.66	0.097	0.06	1.27
Intraoperative Complications	1.09	1.05	0.09	0.927	0.16	7.21

* Significant at *p* < 0.05; Time at risk = 49.69. All patients were monitored 30 days after surgery and follow-up took place in the present year; no further deaths have been recorded.

## Data Availability

The original contributions presented in this study are included in the article. Further inquiries can be directed to the corresponding author.

## Abbreviations

The following abbreviations are used in this manuscript:

ICU	Intensive Care Unit
CID	International Statistical Classification of Diseases
RACHS-2	Congenital Heart Surgery second edition
